# Dignity at the Workplace: Evolution of the Construct and Development of Workplace Dignity Scale

**DOI:** 10.3389/fpsyg.2019.02581

**Published:** 2019-11-29

**Authors:** Anjali Tiwari, Radha R. Sharma

**Affiliations:** Management Development Institute, Gurgaon, India

**Keywords:** dignity at the workplace, dignity measure, scale development, workplace dignity, dignity

## Abstract

Concerns about workplace dignity (WPD) have long driven researchers and practitioners to explore ways of measuring it. It is essential for organizations to understand, how employees perceive the WPD for positive employee outcomes. The paper reviews literature, traces the development of WPD and finds the gap. The purpose of this paper is to evolve and operationalize the construct of ‘WPD,’ and develop and standardize a measure for it which will pave the way for future studies to empirically test the role of WPD on organizational outcomes such as employee engagement, retention and the like. This is perhaps the maiden attempt for conceptualization and operationalization of the construct of WPD, thus it contributes to the extant knowledge and has implications for academics and practitioners.

## Introduction

Workplace dignity (WPD) is a multidimensional concept and is receiving considerable attention due to paucity of research and non availability of a measure to quantify it. This has provided impetus to undertake this study and develop and standardize a measure to assess WPD. The paper traces the evolution of the construct of ‘dignity’ and develops an empirically evolved definition of ‘WPD’ which has considerable significance for organizational outcomes.

The workplace environment plays a major role in an employee’s life because people spend most of their day-time at the workplace. Work performance and feedback can sharpen their capabilities and competencies; therefore, the workplace plays a major role in the capability enhancement (Lucas, 2015). Workplace could also be seen as a facilitator or inhibitor for the development of the potential of an employee. At the workplace employees share a dyad relation hence co-worker behavior, remarks of the superior, recognition, respect, trust are a few variables which have a direct influence on one’s WPD experience. All the remarks and above-mentioned factors may enhance employees’ experience of the WPD whereas their absence may do the contrary, but there is knowledge gap as this requires empirical evidence. The paper endeavors to bridge the knowledge gap.

Dignity is illustrated as somewhat profane and sacred, newfangled and ancient, changeable and absolute, measurable and measureless (Kovach, 1995; Edlund et al., 2013). Moreover, being consistent in nature, dignity can also be described as relative and changeable which has an internal and external side, that could be experienced in relation to someone, or something (Edlund et al., 2013). The changeableness is considered as one of the characteristics through which dignity can be smashed although it could also be restored. Experience of dignity, like the feeling of value, requires that there is somebody who understands and recognizes these values and shows respect for these values (Kovach, 1995). Many scholars explain dignity as a social phenomenon, which could be developed and created by culture, society and by external qualities (Miller, 1997; George, 1998). Also, education and culture play a significant role in the development and understanding of dignity which could be measured through position and characteristics in relation to someone or something (Kovach, 1995; Miller, 1997).

### Typologies of Dignity

**FIGURE 1:**
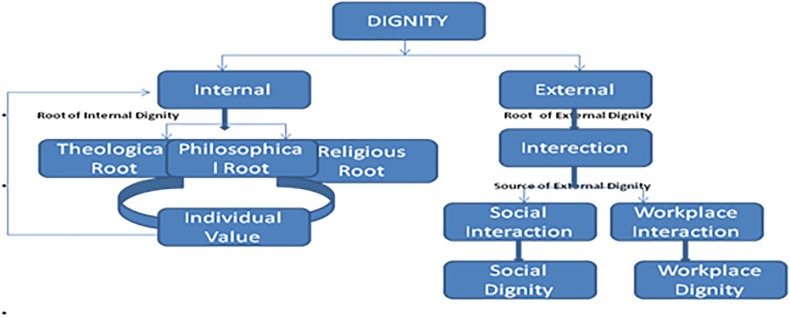
Conceptualization of Dignity. Developed by Authors based on the work of Hodson (2001), Rayman (2001), Sennett (2003), Ackroyd (2007), Bolton (2007), Sayer (2007), Wood and Karau (2009), Crowley (2012), Lucas et al. (2013), Crowley (2014), and Lucas (2015).

Few scholars have classified it into two types- internal (Kim et al., 2010) and external dignity (vide Figure 1) Castel (1996) and Sayer (2005). Internal dignity is described as a gift of God that no one can take from us; we are the sole owner of internal dignity. Dan-Cohen (2011) described that theology and philosophy are the major sources of internal dignity. The main source to experience external dignity is interaction with the outer world. The external dignity, represented by symbol of the values, is shaped by actions, events and individuality. It could be impacted by others’ judgment, one’s productivity and other types of contributions which a person makes to the outer world. The present study did not draw a line between the two types of dignity. For enhancing the conceptual argument Mattson and Clark (2011) in their work raised a logical question, if internal dignity is enough for human flourishing then what is the need for policies and rights to protect human dignity. Based on this understanding the whole world should be the happiest place for work; but the reality is far from this, as interaction and communication are the major part of our daily social life. Dignity develops through interactions with the outer world and this interaction impacts our internal dignity as well. Therefore, both external and internal dignity is connected with undefined lines. However, the explanation of this relation is difficult, yet the denial is also not possible. The literature explains that both internal as well as external dignity is determined by even one’s personality (Acevedo, 2012; Kawamura et al., 2013). Society and surroundings have impact on personality hence social dignity is determined by the harmony, reciprocity, and relationship in a two-way process which cannot exist in isolation. Hence, to get greater insight about the construct a review of the literature on dignity has been presented in the following paragraphs.

### Evolution of Dignity in Management

Dignity as a concept is deep-rooted and dates back to centuries of philosophical and sociological research. Many other classical sociologists and political theorists like Max Weber, Kal Marx, and Emile Durkheim explain the dignity based on the current situation of workers. Karl Marx talked about the denial of WPD. Marx explained exploitation of workers by the capitalist causes alienation from work resulting in the denial of dignity (Healy and Wilkowska, 2017). Bottomore and Rubel (1963) posits that in the absence of meaningful work, workers’ capacity for development gets robbed by capitalist. This situation arises due to the capitalists treating workers as the means of production, and as an inanimate factor of production (Marx, 1971). Durkheim believed both the economic and work life is in a chronic state which he described as “Anomie” or normlessness (Hodson, 2001). Both Emile and Mark have a similar view that capitalism introduces denial of dignity at the workplace. According to Weber excessive rationality and bureaucracy cause the breakdown of norms which cause dehumanization at the workplace (Hodson, 2001).

During the mid of 19th century human relations movement gained favor among management scholars. The main focus of this movement was to put humans into the center and develop all the practices for their development. Thus by that time the concept of ‘human’ gained its real meaning, dignity was the central theme for all the management movements but the biggest irony is it was neither explicitly defined nor became the central theme for discussion (Bolton, 2007; Lucas, 2015). Hodson’s (2001) work enhanced the level of inquiry; in his work, he took ethnography as a methodology and covered a large section of workplaces such as hospitals, factories, restaurants, among others. The major finding was several work conditions providing experience of dignity or denial of dignity. Dignity is a multidisciplinary research topic with the vast coverage of management, sociology, political science, medical science and many more.

### Theoretical Underpinnings

There are two theoretical lenses-. (i) social identity theory (SIT) Tajfel and Turner (2004) (ii) the affective event theory (AET) proposed by Weiss and Cropanzano (1996). The former theory explains the relationship and identity at the workplace, and the latter explains that workplace events shape employees’ emotions and attitudes in positive or negative direction which later lead to dignity or denial of it.

Social identity theory revolves around the individuals and their self- esteem which got influenced by the membership either in the workplace or in society (Ashforth and Mael, 1989). It explains that individuals’ identity is based on their social groups (Stets and Burke, 2000). However, both the above mentioned theories could partially explain WPD, because WPD is a complex concept and so far no single theory could explain it. SIT only considered the identity and relational part; however, AET considered only the events and its impact on the individuals. None of the above mentioned theories could fully explain the dimensions of WPD.

#### Dignity at the Workplace

Dignity has its roots in sociology and management studies, yet its description in the literature is loosely covered by many contributors (Kornhauser, 1964; Freidman, 1977; Ryan, 1977; Rosow, 1979; Schumacer, 1979; Khan, 1981; Agassi, 1986; Fox, 1994; Gini, 2001; Hodson and Roscigno, 2004). In view of the importance of WPD, International Labor Organization (ILO) 1944 in its constitution explained dignity as one of the fundamental human rights and asserts that

“*All human beings, irrespective of race, creed or sex, have the right to pursue both their material well-being and their spiritual development in conditions of freedom and dignity, of economic security and equal opportunity*”.

It is a complex phenomenon Bolton (2010) which has mainly two-fold perspectives - one is denial and another one is the affirmation. The majority of the WPD literature focuses on the denial perspective like mismanagement, overly long hours, bullying and harassment (Wright and Smye, 1997; Ishmael, 1999; Hodson, 2001; Rayman, 2001; Rennie Peyton, 2003). Some scholars talked about developing cultures of respect as a strategy to enhance WPD (Wright and Smye, 1997; Ishmael, 1999; Rennie Peyton, 2003). Though, Lucas (2015) has done some work on the affirmation of dignity, yet there is a gap about how dignity could be defined. Though the importance of dignity has been highlighted, dignity as a concept is struggling for a well-accepted definition and how it is being practiced (Sayer, 2007; Lee, 2008). To develop a greater understanding of workers’ conditions both explicitly and implicitly/internal and external dignity could appear as a lens (Bolton, 2007; Sayer, 2007). In the present scenario when employees spend a large portion of their time at work, WPD becomes necessary for realizing the self-worth (Bolton, 2007). But an employee gets hired to perform a mechanical/instrumental role, so the accomplishment of dignity at work often becomes problematic in an employee-employer relationship (Sayer, 2007). Therefore, the employer- employee association constantly will be rife with possible indignities. A number of recent researches have focused on how individuals’ WPD has been threatened at the workplaces (Crowley, 2012, 2014; Lucas et al., 2013; Lucas, 2015). Steimel (2010) explained the denial of dignity of women workers through the “abusive communication” and “questions on their competence” from seniors and clients. The achievement of WPD could be influenced by words, deeds, and material conditions (Sayer, 2007). Brennan and Lo (2007) have expressed concern over the system that dignity withdrawing practices can turn into social institutions and structures. Hodson (2001) has classified dignity-diminishing practices enhancing the experience of denial of dignity at work into four categories: (i) *mismanagement and abuse*, (ii) *incursions on autonomy*, (iii) *overwork* and (iv) *contradictions of employee involvement*. Edlund et al. (2013) have identified spiritual, soul and physical dimensions as the sources of human dignity but have ignored WPD. Based on the premise that people spend a large portion of their time at the workplace which affects their dignity and with advancing technologies organizations are becoming more mechanistic than humanistic.

#### The Knowledge Gap in Conceptualization and Methodology

Most of the studies have used the qualitative technique as a methodology (Brennan and Lo, 2007; Steimel, 2010; Edlund et al., 2013; Lucas, 2015). Bolton (2010) says, ‘to date there are only limited available insights into what dignity at work might mean to workers and managers in their day-to-day working lives [and] how this impacts upon their experiences of work” (p. 161). Instead, dignity research tends to draw upon researchers’ a priori assumptions, retrospective interpretations, and/or outsider judgments of dignity (see Lucas, 2011; Khademi et al., 2012; Baker, 2014). The biggest irony is scholars like Bolton (2007), Mattson and Clark (2011) have described dignity as a concept but WPD still needs a comprehensive definition. Dignity in management and workplace has been studied by a few researchers in the west (Hodson, 2001; Sayer, 2007), yet there is lack of conceptualization on what exactly WPD means and also there is no concrete definition available though it is observed that the lack of WPD affects performance, productivity and leads to attrition. Lucas (2017), in her recent work, identified the methodological gap and mentioned “*Researchers should develop theoretically and empirically sound measures of dignity in the form of survey scales, inventories/indexes, assessment tools, and so forth”(pp.11).* In the absence of a measure for WPD we have tried to empirically evolve the construct WPD and also developed and standardized a measure for it. Our paper will evolve the operational definition of WPD through the empirical research.

## Materials and Methods

Researchers like Brennan and Lo (2007) and Lee (2008) have highlighted the role of context in understanding of WPD and also the differences between Indian and Western concept of WPD. Following Simms and Watson (2007) and DeVellis (2012) we carried out an extensive literature review on WPD and found limited research work focusing on WPD specifically in the Indian context. The development of WPD scale needs a lot of ground work, Giddings and Grant (2006) advocated the use of multiple methods when studying a less explored complex issues. Accordingly, we incorporated mix methods and use qualitative method as a first step to develop the basic understanding of the construct to be empirically evolved. Based on literature review and qualitative data an exhaustive list of items was generated.

### Phase I: Qualitative

#### Participants and Design

The ambit of this part of research is to understand the phenomenon from practitioners’ perspective and also to utilize the output of the interview analysis for item generation for a pilot study. For the qualitative study managers with 15 plus years of experience from different industries/organizations were targeted for the semi structured interviews. WPD is a dynamic concept which has many dimensions hence to enhance its understanding a wide range of work experience was considered important. For selection of interviewees an invitation mail was sent to 23 managers who fulfilled preliminary selection criteria of experience and occupation. Only 18 managers (10 males and 8 females) gave consent and were selected for the interview. Out of 18 managers, 12 had about 20 years of work experience remaining 6 were had about 15 years of work experience and were in the senior position in their respective organization. Their education level ranged from bachelors, to doctorate level in multiple disciplines. Confidentiality was considered critical due to the sensitive nature of the topic and the importance of obtaining candid and honest response. The participants were assured that all the information collected would be used only for research.

Based on the protocol suggested by Lucas (2017) an interview protocol was developed (Appendix 2) to eliminate any presumptions or bias. After establishing rapport the interviews were conducted following the developed protocol, however, to get an insight into WPD open-ended questions were also asked. WPD is personal and sensitive subject at the workplace in the Indian context hence getting the genuine response of the participants was necessary. The open-ended questions included statement: “How do you explain the concept of dignity; please answer the questions in as much detail as you can.” We decided to keep the first question as general; the idea behind was to understand how managers perceived dignity. Rabionet (2011) mentioned that the opening question of the semi-structured interview should be general. Other researchers supported this order as ‘non threatening to the threatening’ (Weinberg, 1996, 85; Leech, 2002). The average time allocated for each interview was 35 min.

#### Interview Analysis and Item Generation

The qualitative data was collected through interview procedure recommended by Miles et al. (2013). After every interview the whole narrative was transcribed and after reading and re-reading of the transcript responses were coded. The transcripts were carefully scrutinized, and important portions were underlined. The motive behind this process was to identify a theme emerging out of interview data. This process was iterated 18 times and on the completion of the interviews all the transcripts and the themes were re-scrutinized to avoid overlap or ambiguity. The themes that emerged from interview data were: equal opportunity, equality, self-esteem, self fulfillment, respect and positive regards. However, some respondents mentioned about the relevance of trust in WPD.

Thus for generating the items the themes that emerged from interview data plus those appearing in literature were considered. The themes which frequently appeared in the literature were autonomy and freedom (Brennan and Lo, 2007). Thus an initial pool of 30 items was generated in English. The prime focus of item generation was to present a catalog of items that will be used for developing the preliminary test.

#### Item Review

The purpose of this phase was to review the items’ content validity (Schriesheim et al., 1993). Hence 6 experts were identified to review the initially developed list of items. This diversified team of experts comprised of academicians, HR managers and consultant with more than 15 years of work experience in their respective fields. A formal mail was sent to all the experts for seeking their consent; thereafter the initial developed items were sent to them (Presser and Blair, 1994; Willis et al., 2000; Demaio and Landreth, 2004). Another mail was sent to all the 6 experts with detailed instructions to rate the items on coverage, clarity and relevance (DeVellis, 2012). On the basis of ratings received from the experts the items were re-assessed on relevance, coverage and complexity for vocabulary, missing information, possible doubts or length (Sharma Radha and Sharma, 2015). After analyzing the experts’ comments 10 items were deleted as these scored low on relevance and coverage and the test remained with only 20 items after the first attempt for content validity by the experts.

#### Pilot Testing

After the experts’ ratings pilot testing was done on a random sample (*n* = 100) both males and females (55% males; 45% females, in the age range 31–70). The sample of pilot study poised working people with minimum 3 years of work experience. An introductory page covering the objectives of the study with instructions for filling up the questionnaire was added to the questionnaire. Personal survey method was used for data collection during pilot testing. Voluntary participation was invited and anonymity and confidentiality were assured while recognizing their association with the research. The objective of the pilot testing was to develop basic understanding of the latent construct of WPD. At this phase, a metric with six response categories without any neutral point was adopted for data collection on the items. Eluding a neutral selection removes the “easy way out” (Churchill and Iacobucci, 2005) rules out participants from deliberately seeking a non-definition (Cox, 1980) and limits them to opt a choice. Commonly scales with a neutral point are less reliable than those without (Churchill and Peter, 1984). Research findings suggest people tend to opt for neutral or middle option if they could opt it (Bishop, 1987; Garland, 1991; Johns, 2005). Chang (1994) used a model approach to evaluate 4- and 6-point scales after fitting empirical data and concluded that the scale points had no effect on criterion-related validity. Research has also shown that there are no differences among 4-, 5-, 6-, and 11- point Likert scales in terms of mean, SD, item– item correlation, item–total correlation, reliability, exploratory factor analysis, or factor loading (Leung, 2011).

#### Procedure

The data from pilot study (*n* = 100) was put through SPSS 22 to examine the credibility of the preliminary questionnaire. All descriptive statistical values including standard deviations (SD), correlations (r), reliability (I̧) and the mean (M) values were assessed. As for validation, reliability is an essential pre-condition (Nunnally, 1978), therefore, before ascertaining the reliability of the test a principle component analysis was done to check the factor loading of the items. The deletion of the following 2 items resulted in considerable improvement of the reliability coefficient: (1) In my view, respectful interactions pull out bullying and harassment behavior out of the organization, (2) I believe respect enhances the level of self-dependence among employees. At this stage reliability was checked through Cronbach α; the value 0.81 was obtained which is statistically significant (Hinkin, 1995).

#### Validation and Psychometric Properties of the WPD

To understand the latent structure of the measure exploratory factor analysis (EFA) was carried out. Later, confirmatory factor analysis (CFA) was also carried out, and indicators for validity and psychometric properties were assessed.

### Phase II

#### Sample and Data Collection

A sample of 650 employees was drawn by random sampling from public, private and government sector organizations having country wide presence in India through mail survey. The sample was drawn from service sector representing banks, IT, ITes and telecom companies based on work experience of 3 years and above so that they had exposure to WPD. Out of the total questionnaires circulated 550 valid questionnaires were received, the return rate being 84.6%. The analysis of the primary data revealed the gender ratio, 56% males and 44% females, 45.5% responses from private sector, 27.3% from government and 27.2% from public sector organizations which indicated representativeness of the sample. The education level of the respondents was bachelor’s degree: 45.5%: masters 45.5% and doctorate degree 9.1%. The age ratio was 29.25% respondents between 26 and 30 years, 30.15% between 31 and 35 and 40.60% between 36 and above.

#### Data Analysis

For analyzing the data statistically, SPSS- 22 software was used. In the initial stage Barlett’s test and the Kaiser-Meyer-Olkin (KMO) were conducted to see if the collected data were suitable for an EFA (Bartlett, 1954). When the KMO value is greater or equal to 0.60, EFA can be performed (Tabachnick and Fidell, 2007). High values signify a good correlation among the items, which explains that EFA is suitable (Kaiser, 1974). We used factor analysis and CFA as a procedure for empirical validity. EFA with principal component and Kaiser criterion and Varimax rotation were used to identify significant components (Nunnally, 1978; Jackson, 1991). Cronbach’s alpha (Cronbach, 1951) and composite reliability were taken into consideration to assess the internal consistency. Good reliability requires, both values should be equal to or greater than 0.6 (Fornell and Bookstein, 1982). In order to determine the factorial structure of the WPD in sample 2, the correlation matrices were obtained (Llinares-Insa et al., 2018).

The model creation phase started by fitting the initial model to the data (Jöreskog and Sörbom, 2006; Llinares-Insa et al., 2018). For assessing the model fit absolute (Jöreskog and Sörbom, 2006) relative indices were used (Marsh et al., 1996): (a) the Comparative Fit Index (CFI) and the Normed Fit Index (NFI) with a cut-off criteria of greater or equal to 0.08 to 0.90 (Hu and Bentler, 1999); and (b) the Root Mean Square Error of Approximation (RMSEA), with values greater or equal to 0.08 indicates good fit (Hair et al., 2006). CFA was also performed. To estimate the CFA, a baseline model was taken under consideration which derived from theoretical consideration and the EFA.

After getting the dimensionality of the questionnaire, the psychometric proprieties were assessed (criterion validity and reliability). To estimate discriminate validity average variance extracted (AVE) and maximum shared variance (MSV) were calculated (Fornell and Larcker, 1981).

## Results and Discussion

We estimated the means and standard deviations (descriptive statistics) on the 550 sample (see Table 1). The item correlation values were, sufficient. EFA was performed to observe the dimensionality of the scale. The outcome value of KMO was 0.854 (>0.6), and the significance level was 0.01 which indicated that the data were appropriate for performing factor analysis (Hair et al., 2006). Bartlett’s Test (*p* < 0.001) confirmed the fitness of the data for factor analysis (Kaiser, 1960).

**TABLE 1:** Item statistics for workplace dignity scale.

**Item no.**	**Mean**	**SD**	**Communalities**
TR2	5.16	1.07	0.584
TR3	5.17	0.95	0.550
TR4	5.48	0.86	0.727
TR9	5.04	1.12	0.392
EQ5	4.18	1.57	0.623
EQ6	4.27	1.49	0.621
EQ7	4.04	1.53	0.697
EQ8	4.97	1.19	0.352
FT12	4.96	1.23	0.723
FT13	5.06	1.09	0.718
FT14	5.00	1.10	0.747
AU15	4.94	1.12	0.503
AU16	5.05	1.02	0.756
AU17	5.10	1.02	0.651
AU18	5.05	1.21	0.376
SE1	5.53	0.870	0.707
SE11	5.13	0.976	0.527
SE12	4.96	1.23	0.723

The EFA performed with the 18 items showed a consistent internal structure and explained variance was 41.9% for all the factors. Total number of factor which churned out was five with Eigen values >1, following Kaiser’s criterion. Table 2 presents the factor loadings that exceeded 0.40 for the five-factor model. With the help of expert consultation and literature review all the five factors were suitably labeled. Average extracted communalities were near to 4, which indicate that there was good fit with the factor solution. Because the factor loading was equal or greater than 0.4 hence we have all ground to retain all the 18 items in this phase.

**TABLE 2:** Factor loadings for dignity at the workplace scale.

**Item no**	**Trust and respect**	**Equality**	**Fair-treatment**	**Autonomy**	**Self- esteem**
TR2	0.718				
TR3	0.760				
TR4	0.672				
TR9	0.526				
EQ 5		0.702			
EQ 6		0.764			
EQ 7		0.862			
EQ 8		0.534			
FT12			0.729		
FT13			0.700		
FT14			0.808		
AU15				0.639	
AU16				0.734	
AU17				0.713	
AU18				0.421	
SE1					0.593
SE10					0.705
SE11					0.605

**FIGURE 2:**
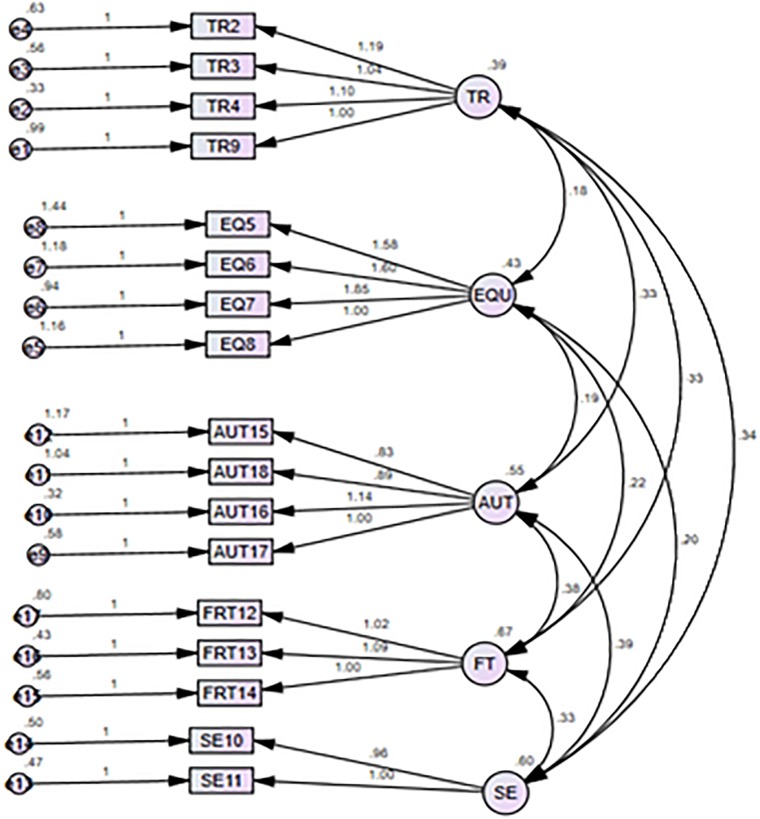
Five Factor Structure of Workplace Dignity derived after confirmatory factor analysis.

Cronbach α value was 0.866 which yields that items of the scale as reliable and scale reliability is of acceptable level. However, we decided to recheck Cronbach α for each item with the option, if item deleted. Thereafter, we performed Cronbach α of the entire test and realized removal of 1 item (SE1) resulted in an improvement of the reliability coefficient (0.868 refer Table 3) (Cronbach, 1951). Although deletion of one item did not greatly impact overall scale’s Cronbach’s alpha value but it was necessary because this item was showing item- total correlation value as 0.191 which was considerably lower than that of the other items. After determining the Cronbach α 0.868 we obtained 17 out of 18 items for the CFA. Van Prooijen and Van der Kloot (2001) stated that the use of CFA is appropriate for validating factor structure. Meyers et al. (2006) described CFA as a deductive process because of the priori specifications that must be made. These specifications include the number of factors, item-factor loading pattern, and correlations between factors (Brown, 2006; Meyers et al., 2006). The measurement model for “WPD” confirmed the five factor structure comprising 17 items (Figure 2). In the initial (one-factor) model, the ratio of X2/df was 4.13, which was under the accepted cut-off value of <5.00. This model indicated a good fit to the data. Next, an inter-correlated five-factor model was specified (see Table 2), agreeing with the results of the EFA study (Llinares-Insa et al., 2018). This model also presented a satisfactory fit. Additionally, it presented reasonable RMSEA values of 0.07 indicating reasonable fit. The measurement model showed a good fit with acceptable values for model fit indices: comparative fit index (CFI) = 0.910, root mean square residual (RMR) = 0.07, root mean square error of approximation (RMSEA) = 0.07, NFI (= 0.866), GFI = (0.926).

**TABLE 3:** Factor-wise Cronbach’s α coefficient.

**Construct**	**Measurement Items**	**Cronbach’s alpha coefficient**	**Composite reliability**
Trust and Respect	TR3, TR2, TR4, TR9	0.739	0.757
Equality	EQ5, EQ6, EQ7, EQ8	0.751	0.760
Autonomy	A15, A16, A17, A18	0.701	0.739
Fair treatment	F12, F13, F14	0.785	0.788
Self esteem	SE10, SE11	0.704 (Before deletion of (SE1) Alpha was 0.562)	0.704

In view of the foregoing the newly developed scale is reliable and valid to assess the dignity of the employees at the workplace. Factors of this scale are conceptualized as noted below.

**Table:** 

**Definition of each factor of Workplace Dignity**	
**Factor**	**Definition**
**Trust and respect**	Individual’s perception about how one is being respected and trusted at the workplace.
**Equality**	Individual’s perception about getting equal treatment at the workplace.
**Self-esteem**	Individual’s perception about one’s worth or self value being perceived at the workplace.
**Fair treatment**	Individual perception about any discrimination, injustice or unfair treatment at the workplace.
**Autonomy**	Individual perception about one’s freedom of expression and decision making at the workplace.

Dignity, as a concept, is commonly associated with worth, value and autonomy (Sayer, 2007). When employees have autonomy to take decisions about how work is to be done, they take pride in accomplishing a job. Researches on individuals done by earlier researchers have focused on only one or two aspects- autonomy, freedom (Bolton, 2013; Lucas, 2015) whereas the present study has empirically evolved a five-factor structure of the construct of dignity.

### Reliability and Validity

For analyzing the reliability of newly developed scale we relied on the value of the Cronbach’s alpha and a composite reliability index of the each indicator within the dimension to which it belongs (in terms of the factorial loadings).

For testing the validity of WPD scale we relied on three aspects. (a) content validity, which reveals whether indicators associated to a construct are representative of the domain they are supposed to measure; (b) convergent validity, which denotes whether different indicators measuring the same concept are highly intercorrelated; and (c) discriminant validity, which refers to the distinctiveness of the factors measured by different sets of indicators. The content validity was of a key concern (Schriesheim et al., 1993) for which the estimation of content validity was based on the researches and subject matter experts. We used both the methods as the item generation was done on the basis of literature and later the 6 experts went through the list of items to see their relevance and coverage for the construct of WPD. Use of this method allowed us to confirm the content validity of the WPD scales. To test the convergent validity of the present scale, the Bentler-Bonett coefficient (BBNNFI) was analyzed. Values near to 0.9 signify the convergent validity of the WPD scale. As scholars like Bollen (1989), advocate seeing the factor loadings as further analysis of convergent validity. Considerable factor loadings also replicate the convergent validity of a scale. The present scale fulfills all the above mentioned criteria; hence we conclude that the present scale shows convergent validity. To test the discriminant validity we checked the value of AVE and Max shared variance (MSV) (see Table 4).

**TABLE 4:** AVE/CR and MSV value.

	**CR**	**AVE**	**MSV**
AUT	0.730	0.519	0.479
FT	0.788	0.555	0.377
EQU	0.760	0.514	0.165
SE	0.704	0.543	0.486
TR	0.757	0.511	0.486

## Discussion

The current study broadens the scholarly work on WPD in significant ways. So far most of the work on WPD has focused on its denial side but not on the achievement or the ways to improve the WPD. The scale developed through the study describes the ways to achieve the employee dignity at work. The importance of WPD not only contributes to employee well-being only but also to the organization overall performance. The final version of this scale consists of 17 items (see Appendix 1) under five dimensions.

### An Empirically Derived Model of WPD and Its Definition

**FIGURE 3:**
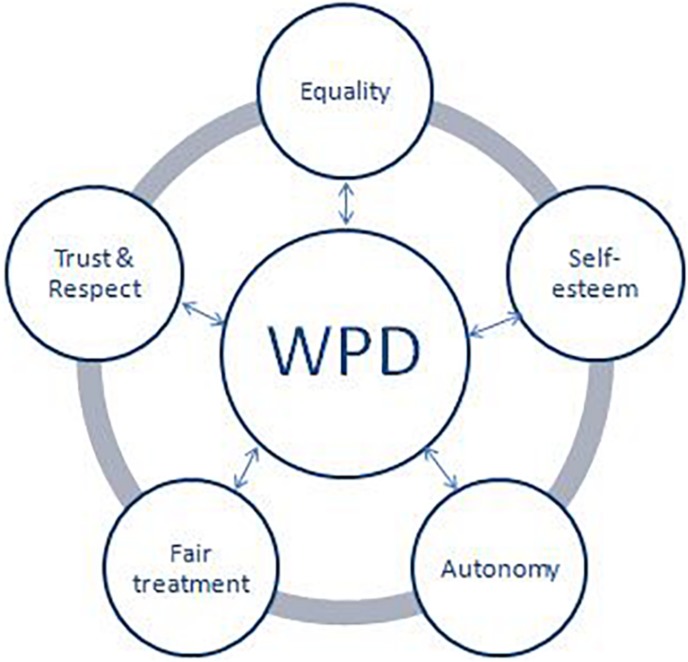
A model of workplace dignity.

This study contributes to conceptualization of WPD as a multidimensional construct. The findings of the study can contribute to introduction of WPD practices which would increase positivity in the organization. This study also developed a model of WPD (Vide Figure 3) which would increase positivity in the organization and will reduce conflict among and between the employees which are expected to increase the dignity experience of the employees at the workplace (Bolton, 2007). Hodson and Roscigno (2004) have posited that the right blend of organizational – level and job level practices have a direct and positive relation with WPD. The present study offers an empirically derived definition of WPD as

“Workplace dignity (WPD) is defined as individual’s perception about respect and trust, equal treatment, valuation of one’s worth, fair-treatment, autonomy and freedom of expression and decision making enjoyed by an employee at the workplace”.

## Conclusion

The present study adopted qualitative approach to develop a preliminary questionnaire on WPD. The source of data for generating the initial items was previous literature on WPD and in-depth interviews with 18 managers with open-ended questionnaires. During the pilot testing, a set of 100 data were collected and cronbach’s alpha for (reliability) and principal component analysis was taken under consideration to check factor loading which helps in extracting the items for the development of the WPD scale. According to Di Fabio and Gori (2016) a series of exploratory factor analyses (EFAs) is required to verify the factor structure of the newly developed scale. To follow this we conducted EFA twice once at the time of pilot testing with the (sample of 100). In another set, we collected 550 questionnaires and used it for EFA and CFA. Through analysis, we gained five factors (Trust & Respect, Autonomy, Fair treatment, Equality, and Self-esteem). After exploratory factor analyses analysis we conducted the CFA the modified results contained: the value of GFI, NFI, RMR and CFI corresponding to 0.926, 0.866, 0.07, 0.910, respectively, the RMSEA value of 0.07. All the indices reflect a suitable level which indicates the WPD scale has an acceptable fitting degree. The Cronbach’s alpha value indicates the reliability of the scale which is 0.868 along with the value of each latent variables of 0.757, 0.760, 0.739, 0.788, 0.704, the CR value of 0.73, 0.788, 0.760, 0.704, 0.757 respectively also the value of AVE is on or above the acceptance level. Hence the above mentioned details validate the developed test. Furthermore, a strict method was followed which ensured its scientificity and accuracy. Consequently, we can determine that the WPD scale shown a decent level of reliability.

### Limitations and Future Research

The objective of the study was to understand the latent construct of WPD, develop a measure, to operationalize it and provide an empirically derived definition. The present study, to the best of our knowledge, is the maiden attempt to develop and standardize a measure for “WPD,” future studies could be undertaken in other cultural contexts to assess if the construct of dignity is influenced by culture. The WPD measure has been developed and standardized on service industry which could be tested by future researchers on manufacturing and other industries as well. Another limitation is that the sample is not representative of the population.

Future studies can use WPD with other individual and organizational variables to assess its impact on individual or organizational performance.

### Practical Implications

Employees have their own understanding of the concept of WPD and there has been no comprehensive definition of it so far. Dignity is not a word but a lens through which manager can analyze the workplace problems and find appropriate solutions based on the findings of the study. Organization may introduce the concept of WPD in their HR policy and employee handbook to resolve many people related problems in the organization. The WPD measure developed through the study could serve a powerful tool to assess workplace dignity to prevent employee dissatisfaction, promote engagement and employee retention.

## Data Availability Statement

The datasets generated for this study are available on request to the corresponding author.

## Ethics Statement

Since it is a social science study, an ethics approval was not required as per applicable institutional and national guidelines and regulations. The respondents participated in the survey on a voluntary basis and were assured that they could leave the survey at any stage, if they so desired. However, informed consent was sought from respondents when data was collected through personal interview and personal survey. The instructions were included in the cover page of the mailed survey.

## Author Contributions

The study was jointly conceptualized during a doctoral course on Psychometric Testing undertaken by AT under the supervision of RS. AT reviewed the literature to identify gap, developed a preliminary questionnaire, items were jointly drafted, discussed, refined, pilot tested, and analyzed, and did data collection, analyzed the results, and prepared the introductory draft which was redrafted, edited, analyzed, and jointly finalized. Both the authors have made substantial, direct and intellectual contribution to the work, and approved it for the publication.

## Conflict of Interest

The authors declare that the research was conducted in the absence of any commercial or financial relationships that could be construed as a potential conflict of interest.

## Appendix 1

**Table:** Workplace dignity scale.

1-strongly disagree, 2-disagree, 3-somewhat disagree, 4-somewhat agree, 5-agree, 6-strongly agree

1. Trust leads to fair treatment.
2. In my view respect enhances your level of trust among the peer group.
3. I will enjoy working in a peer group where the level of trust is high.
4. I will enjoy working in a group where people respect each other.
5. Organization with no equal gender policy leads to sexual harassment.
6. Discrimination on the basis of gender leads to inequality in the organization.
7. Discrimination on the basis of caste or creed leads to inequality in the organization.
8. When there is a mismatch between my skill set and assigned role, it affects my esteem.
9. Unfair distribution of work hurts me.
10. My self esteem is enhanced if I have freedom to decide the process of my work.
11. Discrimination in the allocation of work puts me off. 12. I will enjoy having the authority to take decisions at my workplace.
13. Unfair treatment of colleagues hurts me. 14. I believe inequality leads to injustice. 15. In my view disagreement with a boss/colleague on some matter is appropriate.
16. Lack of freedom of expression affects my autonomy.
17. Not being allowed to disagree affects my sense of autonomy.

Appendix 2 Sample interview protocol.

1. Tell me about the nature of your current job.
  a. Where do you work? What is your position?
  b. What are your responsibilities?
  c. When did you become a manager? What is the team size which you manage?
2. What does dignity mean to you?
3. I want you to think of a time at work when you felt that you were being treated with dignity. What was that experience?
4. Now, I want you to think of a time when you felt as though you were not being treated with dignity. Please describe that experience.
